# Severe and early quadriceps weakness in mechanically ventilated patients

**DOI:** 10.1186/cc13888

**Published:** 2014-05-23

**Authors:** Isabelle Vivodtzev, Andrée-Anne Devost, Didier Saey, Sophie Villeneuve, Geneviève Boilard, Philippe Gagnon, Steeve Provencher, Mathieu Simon, Richard Baillot, François Maltais, François Lellouche

**Affiliations:** 1 Centre de Recherche de l’Institut Universitaire de Cardiologie et de Pneumologie de Québec, Université Laval, 2725 chemin Sainte-Foy, Québec G1V 4G5, Canada; 2 Univ Grenoble Alpes, Grenoble HP2 38000, France; 3 Inserm U 1042, Avenue des Maquis du Grésivaudan, Grenoble 38043, France

## 

ICU-acquired weakness has been reported in patients with prolonged mechanical ventilation [1], leading to prolonged weaning, poor quality of life after ICU discharge, and high ICU-related cost [2]. Muscle weakness is the primary manifestation of critical illness polyneuropathy or myopathy or both.

Although quadriceps strength has never been objectively quantified in the ICU, we previously evidenced quadriceps muscle weakness by using magnetic stimulation of the femoral nerve in patients with chronic obstructive pulmonary disease (COPD) and this non-invasive technique allows a non-effort-dependent assessment of quadriceps strength [3,4]. Thus, one objective of this pilot study was to evaluate the feasibility of assessing quadriceps strength by using this previously validated technique in sedated patients on mechanical ventilation at different stages after ICU admission using magnetic stimulation of the femoral nerve. The study was approved by the ethics committee of the Institut Universitaire de Cardiologie et de Pneumologie de Québec (CER20392). Signed informed consent was obtained from relatives for all patients.

Quadriceps twitch tension (Twq) assessment was performed in 13 consecutive sedated and mechanically ventilated patients with organ failure (Table 1). Twq measurements were repeated after awakening in nine patients. Mean Twq was 1.8 ± 1.3 kg for the whole group of patients. As shown in Figure 1, Twq was two times lower in ICU patients than in COPD patients (*P* <0.001) and four times lower than in healthy subjects (*P* <0.001). Furthermore, there was no significant difference in Twq when patients were sedated or awake. The reproducibility between these two measurements was good (Figure 2). Strength measurements have been performed in patients during septic shock (n = 2) or after a dialysis session (n = 2), and a major reduction of muscle strength (Twq <1 kg) was observed in these circumstances.

**Table 1:** Patient characteristics at baseline

Demographics	
Males/females, number	8/5
Age, years	71 ± 9
Body mass index, kg/m^2^	25 ± 4
Arterial blood gases	
PaO_2_, mm Hg	82 ± 34
PaCO_2_, mm Hg	41 ± 8
pH	7.43 ± 0.08
SaO_2_, percentage	95 ± 2
ICU admission	
Cardiac surgery ICU	9 (69%)
Respiratory ICU	4 (31%)
Comorbidities	
COPD	8 (62%)
Hypertension	11 (85%)
Hypothyroid	3 (23%)
Dyslipidemia	8 (62%)
Diabetes mellitus	4 (31%)
Risk factor for polyneuropathy	
Mechanical ventilation more than 72 hours	12 (85%)
Suboptimal glucose control^a^	11 (85%)
Steroids	5 (38%)
Septic shock	6 (46%)
Neuromuscular blocker	5 (38%)
Risk factors, mean	3 ± 1
Duration of hospitalization before strength assessment, days	7 ± 4
Sedation condition, RASS score	-3.8 ± 1.5

Data are presented as mean ± standard deviation or as number (percentage). ^a^Suboptimal glucose control is defined as repeated measurements of capillary or venous glucose measurements above 10 mmol/L (at least two consecutives). COPD, chronic obstructive pulmonary disease; PaCO_2_, arterial pressure in carbon dioxide; PaO_2_, arterial pressure in oxygen; RASS, Richmond Agitation-Sedation Scale; SaO_2_, arterial saturation in oxygen.

**Figure 1:**
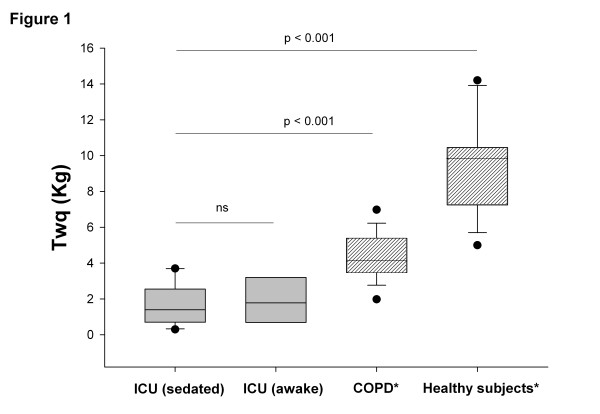
**Quadriceps twitch tension (Twq) in ICU patients.** Stimulation was applied in ICU mechanical ventilation patients who were sedated (n = 13) or awake (n = 7) (grey), patients with age-related chronic obstructive pulmonary disease (COPD) (n = 18), and healthy subjects (n = 16) (shaded). The ends of the boxes define the 25th and 75th percentiles, and a line at the median and error bars define the 10th and 90th percentiles. *Previously measured in our laboratory [3]. ns, Not significant.

**Figure 2:**
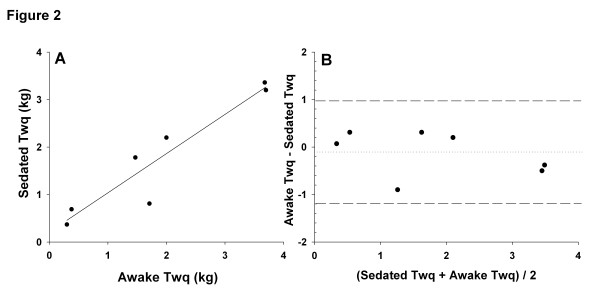
**Reproducibility of quadriceps twitch tension (Twq) measurements in sedated versus awake conditions. (A)** Linear regression between Twq measured in sedated versus awake conditions in mechanical ventilation patients (Spearman coefficient correlation, r = 0.93, *P* = 0.02). **(B)** Bland-Altman comparison of sedated and awake Twq measurements. Limits of agreement (reference range of differences) were -1.18 and 0.98 kg. The means bias was -0.13 kg with a standard deviation of 0.47 kg.

Our results confirm the evidence of early severe muscle weakness in mechanically ventilated patients and show that measurement of muscle strength by magnetic stimulation of the femoral nerve may be useful in ICU patients, particularly for assessing recovery or the effect of therapeutic interventions, as previously suggested by Ginz and colleagues [5]. A noteworthy result is that some events (such as dialysis and sepsis) may modify the muscle strength and need to be considered when interpreting muscle strength data in this context. Our data showing that muscle weakness is an early process in the ICU favor early treatment to prevent rather than delay treatment to treat this condition.

## Abbreviations

COPD: Chronic obstructive pulmonary disease; Twq: Quadriceps twitch tension.

## Competing interests

The authors declare that they have no competing interests.

## Authors’ contributions

IV and FL contributed to the study concept and design, data analysis, the interpretation of results, and the writing of the manuscript. A-AD, DS, SV, GB, and PG participated in the recruitment of patients, data acquisition, and the writing of the manuscript. SP, MS, and RB participated in the recruitment of patients. FM contributed to the study concept and design and the writing of the manuscript. All authors read and approved the final manuscript.
